# A Comparison of the Throat Symptoms of Lachesis and Lycopodium

**Published:** 1893-05

**Authors:** E. V. Ross

**Affiliations:** Rochester, N. Y.


### A COMPARISON OF THE THROAT SYMPTOMS OF LACHESIS AND LYCOPODIUM.

E. V. Ross, M. D., Rochester, N. Y.

In treating a number of cases of diphtheria during the past winter, my attention was called (in endeavoring to find the simillimum for the case in hand), to the similarity between the throat symptoms of *Lachesis* and *Lycopodium*, and in order to aid me in differentiating between the two, I made the following plan of comparison in my interleaved copy of Oehme's *Therapeutics of Diphtheritis*, and thinking it might be of some practical value to others, I herewith submit it.

I would also state, by way of parenthesis, that there was a time when I resorted to swabs, gargles, and sprays, but have abandoned this piece of nonsense, and now trust the case to the simillimum *alone*, with quicker recoveries and a lessened mortality as my reward.

| SIMILAR. | | REMARKS. |
|---|---|---|
| Chronic enlargement of tonsils. | *Lach. Lyc.* | |
| Swelling and suppuration of tonsils. | *Lach. Lyc.* | Lach. gangrenous. |
| Constant desire to swallow. | | Under Lyc. it amounts almost to a spasm. |
| Choking (constricted) feeling in throat. | Lach. *Lyc.* | |
| Much phlegm (mucus) in fauces. | *Lach.* Lyc. | Under Lach. it excites painful hawking, under Lyc. an inclination to swallow. |
| Relief from *cold* drinks. | *Lach.* | |
| Relief from hot drinks. | *Lyc.* | |
| Swallows *solids* more easily than liquids. | Lach. | |
| Swallows liquids more easily than solids (verified). | Lyc. | Mat. Med. gives difficulty in swallowing solids. |
| Pain out of proportion to the amount of swelling. | *Lach.* | |
| Swelling out of proportion to the amount of pain. | *Lyc.* | |

| CONDITIONS. | | REMARKS. |
|---|---|---|
| Asthenic. | *Lach.* | Under Lach. there may be suspicious absence of any pyrexia. |
| Sthenic. | *Lyc.* | Under *Lyc.* we may have a high temperature. |

*General Remarks.*—*Lach.* and *Lyc.* are complementary, especially so in throat troubles. One takes up the work where the other leaves off. If, after you have given *Lachesis,* the membrane goes over to the right side and becomes fixed, Lycopodium comes in as a substitute. If the reverse of this condition, give *Lachesis.* And in my experience the higher potencies (200 to CM) act better than the lower.

| SIMILAR. | | REMARKS. |
|---|---|---|
| Regurgitation of liquids through nose. | *Lach.* Lyc. | |
| Fetor oris. | Lach. *Lyc.* | |
| Aggravation from swallowing liquids. | *Lach.* Lyc. | Lyc. is worse by cold liquids (milk), but better by warm. |
| Aggravation after sleep (even a slight nap). | *Lach.* Lyc. | Lach. the patient sleeps *into* an aggravation; Lyc. is simply aggravated after sleep. |
| DISSIMILAR.* | | |
| Pain, swelling, and deposit begin on the left side. | Lach. | In the *Guiding Symptoms* we also find the following as having been repeatedly verified: "Right tonsil first affected, then the left." "Soreness of throat commencing on right side and passing to left, where it becomes fixed. |
| Pain, swelling, and deposit begin on the right side. | *Lyc.* | |
| Sensation as if a lump descended in throat on swallowing, but returns. | | Lac-can. has exactly this symptom, but worse on the right side. |
| Sensation as if a lump rose up in throat on swallowing. | *Lyc.* | Excites choking. |

*Hom. World,* April 1st. Vol. XXVIII, page 328.

---

*"I am aware that this term, as here applied, is somewhat arbitrary, but have adopted it more for convenience sake. While the symptoms are in some respects similar, they are also in some respects dissimilar."

